# Increased levels of soluble CD226 in sera accompanied by decreased membrane CD226 expression on peripheral blood mononuclear cells from cancer patients

**DOI:** 10.1186/1471-2172-10-34

**Published:** 2009-06-02

**Authors:** Zhuwei Xu, Tao Zhang, Ran Zhuang, Yun Zhang, Wei Jia, Chaojun Song, Kun Yang, Angang Yang, Boquan Jin

**Affiliations:** 1 Department of Immunology, the Fourth Military Medical University, Xi'an, PR China; 2 Department of Neurosurgery, Tangdu Hospital, the Fourth Military Medical University, Xi'an, PR China

## Abstract

**Background:**

As a cellular membrane triggering receptor, CD226 is involved in the NK cell- or CTL-mediated lysis of tumor cells of different origin, including freshly isolated tumor cells and tumor cell lines. Here, we evaluated soluble CD226 (sCD226) levels in sera, and membrane CD226 (mCD226) expression on peripheral blood mononuclear cells (PBMC) from cancer patients as well as normal subjects, and demonstrated the possible function and origin of the altered sCD226, which may provide useful information for understanding the mechanisms of tumor escape and for immunodiagnosis and immunotherapy.

**Results:**

Soluble CD226 levels in serum samples from cancer patients were significantly higher than those in healthy individuals (*P *< 0.001), while cancer patients exhibited lower PBMC mCD226 expression than healthy individuals (*P *< 0.001). CD226-Fc fusion protein could significantly inhibit the cytotoxicity of NK cells against K562 cells in a dose-dependent manner. Furthermore, three kinds of protease inhibitors could notably increase mCD226 expression on PMA-stimulated PBMCs and Jurkat cells with a decrease in the sCD226 level in the cell culture supernatant.

**Conclusion:**

These findings suggest that sCD226 might be shed from cell membranes by certain proteases, and, further, sCD226 may be used as a predictor for monitoring cancer, and more important, a possible immunotherapy target, which may be useful in clinical application.

## Background

CD226, also named platelet and T cell antigen 1 (PTA1) or DNAX accessory molecule-1 (DNAM-1), is a transmembrane glycoprotein belonging to the immunoglobulin superfamily. The CD226 molecule is mainly expressed on NK cells, T cells, NK T cells, and platelets, and is involved in cytotoxicity and cytokine secretion of T cells and NK cells and in platelet aggregation and activation [1-4]. It is highly conserved among human, gibbon, monkey, and mouse, suggesting this molecule may have important biological functions [5]. Recently, CD226 has been identified as a receptor for CD112 and CD155 [6], and ligation of CD226 and leukocyte function-associated antigen-1 (LFA-1) with their respective ligands cooperates in triggering cytotoxicity and cytokine secretion by T and NK cells [7].

Recently, more attention has been paid to a putative role for CD226 in tumor development. The specific interaction between CD226 (on NK cells) and CD155 or CD112 (on tumor cells) plays an important role in the NK-mediated lysis of tumor cells [8-11]. Based on that, CD226 is thought to be one of the major activating NK receptors [12,13] and involved in tumor immunosurveillance [14]. Moreover, the abnormal expression of CD226 and its ligands in some tumors may be involved in the mechanisms of tumor escape, invasion, and migration [15,16]. Although soluble CD155 has been detected in human serum and cerebrospinal fluid [17] and is anticipated to be one of the mechanisms of tumor immune escape [14], there has been no report on soluble CD226 (sCD226) or the levels of membrane CD226 (mCD226) and their relationship in tumors. Here, based on the mAbs and ELISA system established by our laboratory [18], we describe increased sCD226 levels in sera and lower mCD226 expression on PBMC from cancer patients compared with those of normal subjects, and demonstrate the possible function and origin of the increased sCD226. These findings may provide useful information for understanding the mechanisms of tumor escape and for immunodiagnosis.

## Results and Discussion

### Elevated sCD226 levels in sera and reduced mCD226 expression on PBMC in cancer patients

**Figure 1:**
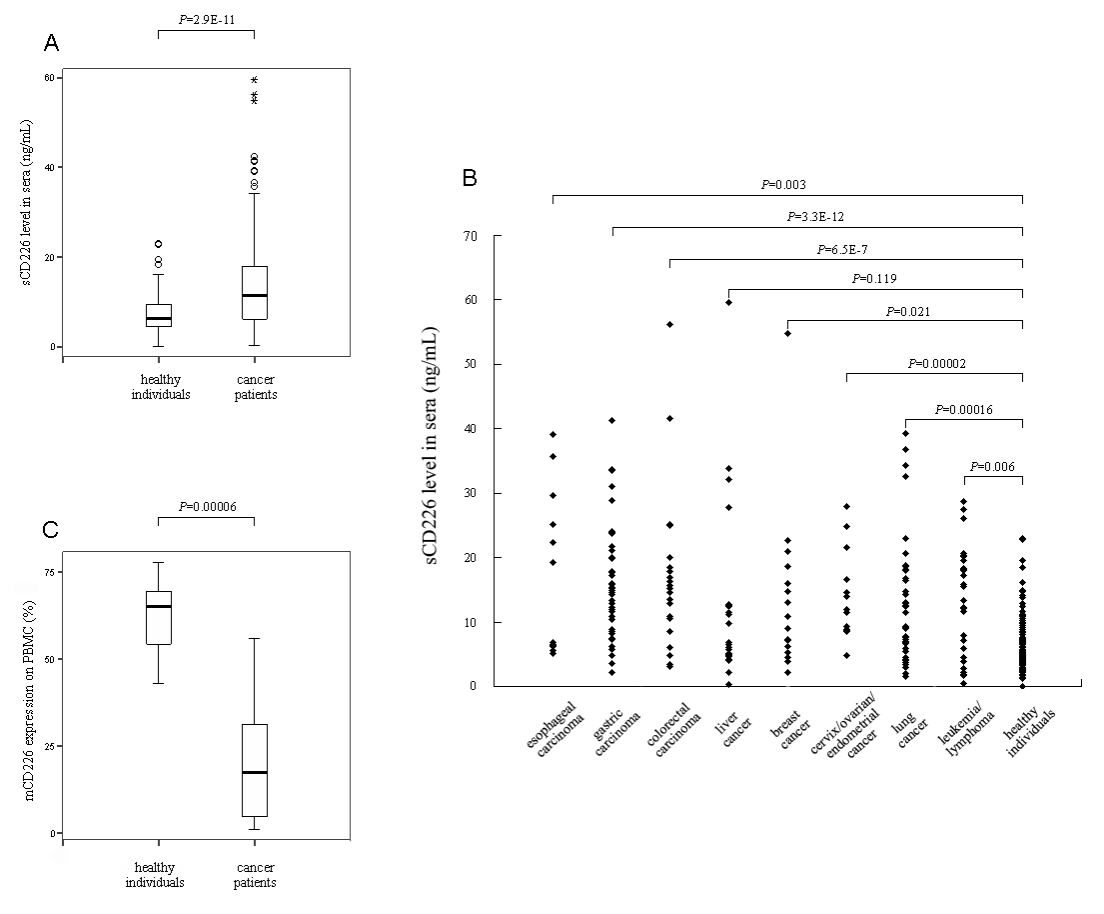
**Sera sCD226 level and PBMC mCD226 expression of cancer patients and normal subjects**. (A) Comparison of sCD226 levels in sera of cancer patients and healthy individuals. Plots (generated using SPSS) show the range of data values obtained. Two hundred and fifty-nine cancer patients (135 male and 124 female, ages 25–65 y) were compared with 129 healthy individuals (69 male and 60 female, ages 25–65 y). (B) Comparison of sCD226 levels in sera of individual patient groups and control group. Data of 49 patients with other kinds of tumors are not shown here due to the large number of tumor types and small sample number of each type. (C) Comparison of positive percentage of mCD226 on PBMC from cancer patients (10 male and 4 female, ages 25–45 y) and healthy individuals (8 male and 4 female, ages 25–40 y). *Top and bottom whiskers*, values of the top and bottom 25% of the cases, respectively; *boxed area*, inter-quartile range and the significant *P *values between groups; *horizontal black line*, median value; *stars and circles*, extreme and outlying values, respectively (as defined by SPSS).

Using the Sandwich ELISA, we found that the concentration of sCD226 was significantly higher in serum from cancer patients than in serum from normal subjects: the ranges of serum sCD226 being 0.1–23 (median 6.3) ng/mL in normal subjects and 0.2–60 (median 11.5) ng/mL in cancer patients (*P *< 0.001, Fig. 1A). This increase in serum sCD226 was evident in most of the tumor types we tested except liver cancer (Fig. 1B), a finding consistent with the fact that all of the tumor types shown here have been identified to express CD155 and/or CD112 [8,17,19-22]. It is known that many membrane molecules can be shed from the cell surface and released into the circulation in soluble form in some pathological conditions such as tumor [23-26]. To investigate whether the increased sCD226 in cancer patients might be shed from the cell surface, we examined the mCD226 on PBMC from both patients and normal subjects. Accordingly, it was found that the expression of mCD226 by PBMC from cancer patients was lower than that from normal subjects, with a statistically significant difference (*P *< 0.001, Fig. 1C). The reduction of mCD226 expression on PBMC from cancer patients was not surprising, since it had been reported that CD226 was reduced on CD56^dim ^NK cells from myeloma patients with active disease compared with patients in remission and healthy controls [11].

### Inhibitory effect of CD226-Fc fusion protein on the cytotoxicity of normal PBMC against K562 cells

**Figure 2:**
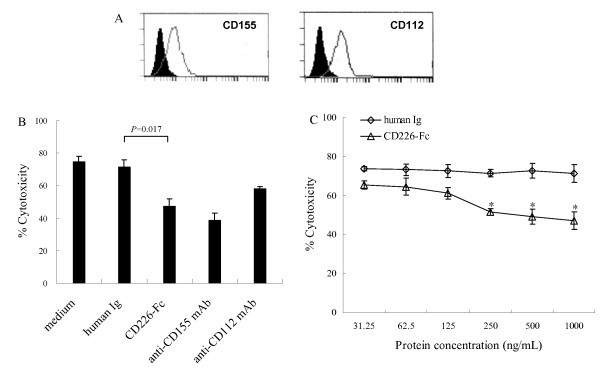
**Effect of CD226-Fc fusion protein on cytotoxicity of normal PBMC against K562 cells**. (A) Expression of CD155 and CD112 by K562 cells. *Solid line*, CD155 and CD112 staining; *black areas*, isotype controls. (B) Inhibitory effect of CD226-Fc fusion protein (1000 ng/mL) on cytotoxicity of NK cells against K562 cells. Medium/human Ig (1000 ng/mL) and anti-CD155/CD112 mAbs were used as negative and positive control, respectively. (C) Inhibition of CD226-Fc on cytotoxicity of NK cells against K562 cells in a dose-dependent manner (*, *P *< 0.05 vs. human Ig of corresponding concentration). The data were the mean ± SD of four wells for each group. Representative experiments are shown (n = 3).

It has been well accepted that the cytotoxicity mediated by NK cells and effector T cells is one of the major mechanisms of immune surveillance against tumor cells. However, malignant cells often have developed strategies that counteract immune surveillance of the hosts, such as secreting soluble activating killing receptors and/or their ligands to avoid or reduce this kind of killing [27-30]. To investigate the possible effect of sCD226 on this cytolytic function against malignant cells, and whether sCD226 could be one of the tumor escape strategies, a CD226-Fc fusion protein was used to mimic the function of sCD226 *in vitro*. NK cytolytic activity was then measured by co-incubation of normal PBMC with the NK-sensitive K562 cell line, which expresses both CD226 ligands (Fig. 2A), in a standard ^51^Cr-release assay, with or without CD226-Fc fusion protein. As expected, CD226-Fc fusion protein could inhibit the cytotoxicity of NK cells against K562 cells in a dose-dependent manner and the inhibition was of statistic significance when the concentration was 250, 500, and 1000 ng/mL (*P *< 0.05, Fig. 2B and Fig. 2C). Although the effective concentration of CD226-Fc required for inhibition *in vitro *was 10–20 fold greater than that observed in patients' sera, the situation *in vivo *is likely to be quite different. First, it is known that many different inhibitory molecules are shed by tumor cells, therefore sCD226 is likely to constitute only one of a number of molecules that each contribute to inhibition; second, natural sCD226 *in vivo *may contain structural differences from the CD226-Fc fusion protein, which was expressed and purified artificially and in a form of homodimer linked by human Ig Fc fragment, and these may yield a higher affinity of binding to CD155 and/or CD112. It can be speculated, therefore, that the increased sCD226 in sera from tumor patients might play a role in blocking or inhibiting the binding of mCD226 to its ligands on the tumor cell, thereby inhibiting tumor cell lysis.

### Regulation of sCD226 and mCD226 by protease inhibitors

**Figure 3:**
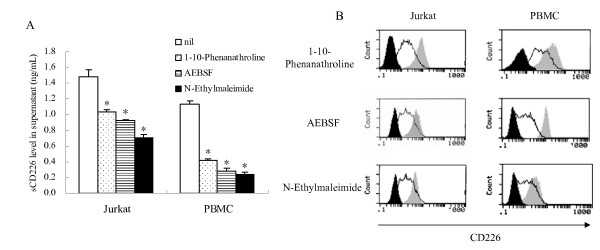
**Effect of protease inhibitors on sCD226 level and mCD226 expression of normal PBMS or Jurkat cells**. (A) Reduced levels of sCD226 in supernatant of PMA-stimulated PBMC or Jurkat cells treated with or without 1 mM protease inhibitor (1–10-Phenanathroline, AEBSF, or N-Ethylmaleimide). The data were the mean ± SD of three wells for each group. (*, *P *< 0.05 vs. nil group). (B) Enhanced expression of mCD226 on PMA-stimulated PBMC or Jurkat cells treated with or without 1 mM 1–10-Phenanathroline, AEBSF, or N-Ethylmaleimide. *Solid line*, without protease inhibitors; *grey areas*, with protease inhibitors; *black areas*, isotype controls. Representative experiments are shown (n = 3).

Having observed the increased serum sCD226 in cancer patients and presenting a possible role for the increased sCD226, we then sought to determine the origin of the sCD226. Based on the fact that many transmembrane molecules are cleaved by certain proteases and shed into the circulation, and that several kinds of proteases can be induced *in vitro *by treatment with PMA [31-33], we investigated the effect on the regulation of sCD226 and mCD226 of PMA-stimulated normal PBMC and Jurkat cells by the treatment with 15 different protease inhibitors (data not shown). As shown in Fig. 3A and 3B, three of the 15 protease inhibitors (1–10-Phenanathroline: a metalloprotease inhibitor; AEBSF: a serine protease inhibitor; N-Ethylmaleimide: a cysteine protease inhibitor) significantly decreased sCD226 level and increased mCD226 expression of the cultured cells treated with PMA. These data indicate that CD226 can be shed from the cell surface by proteolysis and released into the circulation in soluble form, and this shedding might involve more than one protease. Similarly, many other transmembrane receptors and cell adhesion molecules, can be shed from the cell surface by proteolysis and released into the circulation in soluble form, and such shedding can involve more than one protease [34]. Our finding that this process likely occurs in a number of cancers is perhaps not surprising since it is known that in these conditions many proteases are up-regulated [35].

### Characterization of the sCD226

**Figure 4:**
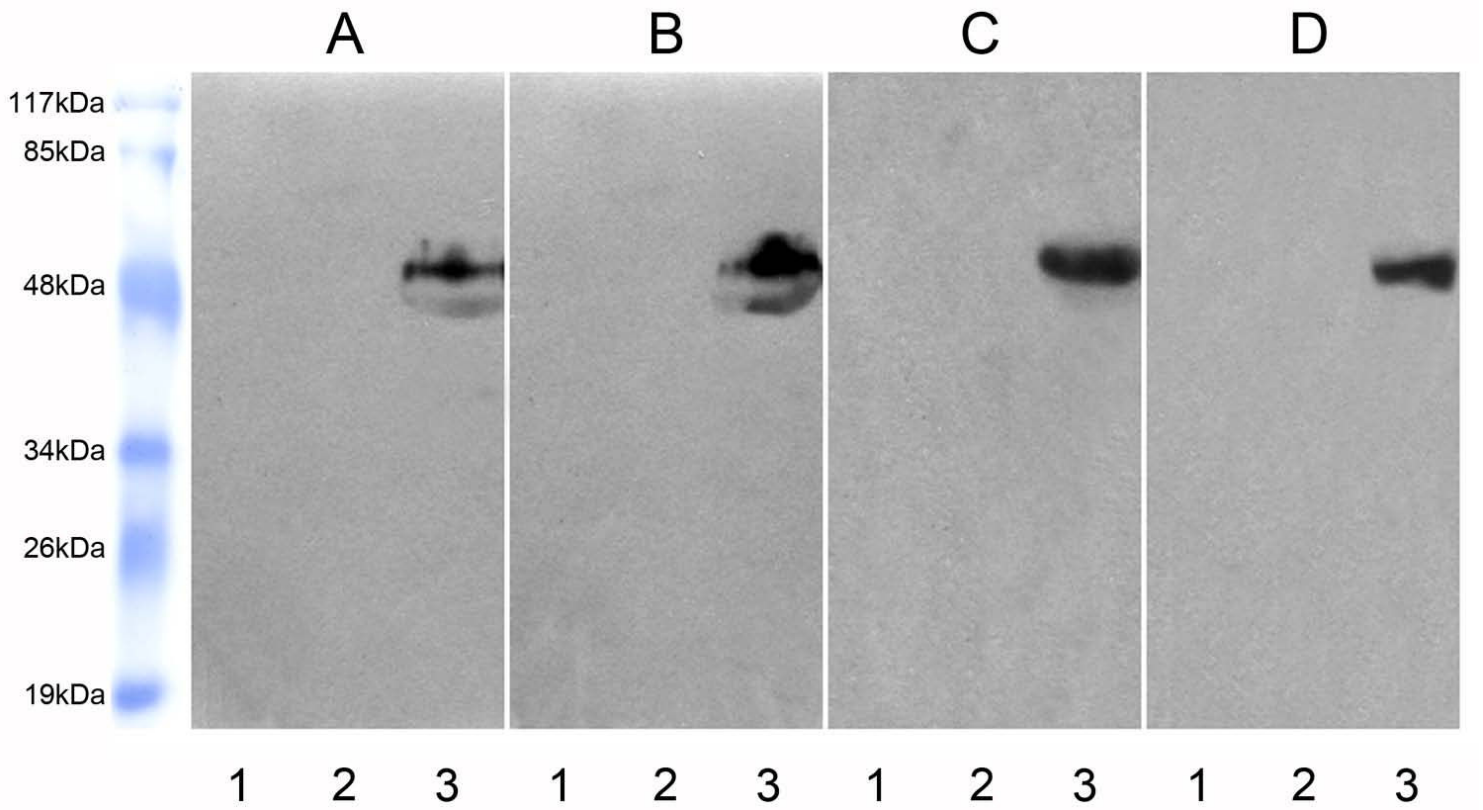
**Characterization of molecular weight of sCD226**. Sera from healthy individuals (A) and cancer patients (B), or supernatant from PMA-activated PBMC (C) and PMA-activated Jurkat cells (D) were precipitated and detected as following: Lane 1, the samples were precipitated with normal mouse IgG-Sepharose 4B (negative control for immunoprecipitation) and detected with FMU4 (anti-CD226 mAb) in Western blot. There was no band because sCD226 could not be precipitated by normal mouse IgG-Sepharose 4B. Lane 2, the samples were precipitated with anti-CD226 mAb, LeoA1-Sepharose 4B and detected with anti-SED (staphylococcal enterotoxin D) mAb (negative control for blotting reagent) in Western blot. There was still no band because the precipitated sCD226 could not be detected with anti-SED mAb in Western blot. Lane 3, the samples were precipitated with LeoA1-Sepharose 4B and detected with the other anti-CD226 mAb, FMU4, in Western blot. There were bands because sCD226 could be precipitated by LeoA1-Sepharose 4B and detected with FMU4 in Western blot. One representative experiment is shown (n = 3).

In order to exclude the possibility that the sCD226 was, in fact, intact mCD226 derived from dead cells, we characterized the molecular weight of sCD226 both in sera samples from healthy donors and cancer patients and in the culture supernatants from normal PBMC and Jurkat cells treated with PMA for 24 h. All the four samples were aliquoted into 3 tubes: one was precipitated with normal mouse IgG-Sepharose 4B and probed with an anti-CD226 mAb FMU4 [18], one was precipitated with LeoA1-Sepharose 4B and probed with anti-SED (staphylococcal enterotoxin D) mAb, and the other was precipitated with LeoA1-Sepharose 4B and probed with FMU4. As shown in Fig. 4, protein bands were only observed when the samples were precipitated with LeoA1-Sepharose 4B and probed with FMU4. As expected, a band with molecular weight of ~50 kDa, coincident with the extracellular domain of CD226 molecule, was found in all the four kinds of sera and supernatant samples. Interestingly, another weaker band, with molecular massed of ~45 kDa, was observed in sera samples, but not in supernatant samples. Based on these findings, we speculate a reasonable explanation for the smaller molecule might be the variety of proteases *in vivo*, leading to the cleavage of sCD226 from different sites of the intact molecule. However, the possibility of heterogeneous glycosylation of sCD226 *in vivo *cannot be excluded.

## Conclusion

We have revealed that the sCD226 levels in sera of cancer patients is significantly enhanced compared with that in normal subjects, while there is a concomitant reduction in mCD226 expression on PBMC isolated from cancer patients. CD226-Fc fusion protein can notably inhibit the cytotoxicity of normal PBMC against K562 cells, indicating that the increased sCD226 might be one of the immune escape strategies used by tumor cells. Three protease inhibitors can decrease sCD226 level and increase mCD226 expression of PMA-stimulated PBMC and Jurkat cells, indicating that the increased serum sCD226 in cancer patients might be shed from the cell surface by several proteases, which are often up-regulated when tumor occurs. These findings suggest that sCD226 might be used as a predictor for monitoring cancer, and more important, a possible immunotherapy target, which may be useful in clinical application.

## Methods

### Patients' and control sera

Samples of patients' sera were obtained from 259 patients with various kinds of tumors, before treatment, within 1 week after entrance to Xijing Hospital, the Fourth Military Medical University. The series included a total of 13 esophageal carcinoma (7 male and 6 female), 45 gastric carcinoma (25 male and 20 female), 24 colorectal carcinoma (11 male and 13 female), 23 liver cancer (13 male and 10 female), 16 breast cancer (female), 15 cervix/ovarian/endometrial cancer (female), 46 lung cancer (28 male and 18 female), 28 leukemia/lymphoma (14 male and 14 female), and 49 others (including tumor in central nervous system, in kidney, in bladder, in parotid gland, in thyroid gland, in submaxilary gland, in suprarenal gland, in prostate gland, cholangiocarcinoma, melanoma, nasopharyngeal carcinoma, laryngocarcinoma, soft tissue sarcoma, and osteogenic sarcoma). Normal sera were isolated from 129 healthy volunteers (69 male and 60 female). Both the patients and the volunteers were 25–65 years old. They had no chemotherapy and had no significant signs or symptoms of microbial infection at the time of entrance. This study was approved by the institutional review board of the University and all individuals provided written and/or oral informed consent. The sera were stored at -20°C until use.

### Sandwich ELISA

100 μL of the anti-CD226 mAb, LeoA1 [1] (2.5 mg/L in 0.05 M sodium carbonate buffer, pH9.5) was added to each well of an ELISA plate (Nunc, Roskilde, Denmark) and incubated overnight at 4°C. After three washes, serum samples or standard CD226 serially diluted with PBS containing 1% BSA and 0.1% (v/v) Tween-20 were added to the wells and incubated for 1 h at 37°C. After extensive washing with PBS containing 0.1% (v/v) Tween-20 (PBS/Tween), the wells were reacted with another anti-CD226 mAb [18] which was conjugated with HRP and diluted in PBS containing 3% PEG for 1 h at 37°C. Color development was performed by adding 100 μL TMB (eBioscience, CA, USA) for 10 min at 37°C and stopped by 2 M H_2_SO_4_. The absorbance at 450 nm was determined with a microplate reader (BioRad, CA, USA).

### Flow cytometry analysis

PBMC were freshly isolated from peripheral blood of healthy adults or cancer patients by Isopaque-Ficoll (Hao Yang, Tianjin, China) gradient centrifugation and incubated with LeoA1 or control IgG1 (BD BioSciences, CA, USA) at 4°C for 30 min, followed by washing and incubation with FITC-labeled goat anti-mouse Ig (Dako, Glostrup, Denmark) at 4°C for 30 min and then analyzed on a FACScan (BD BioSciences). Expression of CD155 and CD112 on K562 cells were analyzed similarly.

### ^51^Cr-release assay

NK cytolytic activity was measured after co-incubation of normal PBMC with the NK-sensitive K562 cell line by a standard ^51^Cr-release assay. Briefly, K562 cells were labelled with ^51^Cr (Amersham biosciences, NJ, USA, 100 μCi/10^6 ^cells) for 2 h at 37°C. After three washes, ^51^Cr-labelled target cells were incubated with various concentrations of CD226-Fc fusion proteins, control protein (human Ig), or anti-CD155/CD112 mAb for 30 min at 37°C. Meanwhile, freshly isolated PBMCs were incubated with 10% of normal human serum or normal murine serum for 30 min at 37°C. Then the target cells and effector cells were pooled together (1×10^4^/well K562 and 1×10^5^/well PBMC) and incubated for 4 h at 37°C. The percentage of cytotoxicity was calculated as percentage specific lysis, as follows:



### Cell culture and stimulation

Freshly isolated normal PBMC or Jurkat cells were cultured in RPMI 1640 (Hyclone, UT, USA) supplemented with 10% FCS, 100 U/mL penicillin/streptomycin, 10 mM HEPES, and 50 μM β-mercaptoethanol at 37°C in 5% CO_2_. The cells (1×10^6 ^cells/mL) were stimulated with PMA (50 ng/mL, Sigma-Aldrich, MO, USA) for 6 h, washed twice, and co-cultured with or without different protease inhibitors (Sigma-Aldrich, INHIB-1 and 131377) for 6 h. Although the concentration of the 15 protease inhibitors was not all the same, the optimized concentration for 3 effective protease inhibitors (1–10-Phenanathroline, AEBSF, and N-Ethylmaleimide) was all 1 mM. Then sCD226 level in the supernatants was detected by the ELISA mentioned above, and mCD226 expression on the cells was analyzed by flow cytometry.

### Characterization of molecular weight of sCD226

Twelve ml of serum from cancer patients and healthy adults, respectively, and 30 ml of culture supernatant from normal PBMC and Jurkat cells treated with PMA (50 ng/mL) for 24 h were used to characterize the molecular weight of sCD226 by immunoprecipitaion and Western blot analysis. Briefly, LeoA1 or normal mouse IgG was covalently coupled to CNBr-activated Sepharose 4B (GE Healthcare, London, the United Kingdom) according to the manufacturer's instructions. After preclearing, the sera or supernatant samples were aliquoted and incubated with 100 μL of LeoA1-Sepharose 4B or normal mouse IgG-Sepharose 4B (negative control for immunoprecipitation) at 4°C overnight. After 4 times washing (20 min incubation for each washing) with Washing Buffer (10 mMTris-HCl, 140 mM NaCl, 0.5 mM MgCL_2_, 0.5 mM CaCL_2_, 0.02% NaN_3_, pH7.4) and following the last centrifugation, the supernatant was aspirated and 40 μl of 2 × loading buffer was added to the bead pellet, vortexed, heated (90–100°C, 5 min), and centrifuged. The supernatant was gently collected, without disturbing the pellet, and then loaded onto 12% SDS-PAGE as the order in Figure 4, and transferred to one complete nitrocellulose membrane (Millipore, Massachussets, USA). The membrane was blocked with 5% (w/v) bovine skim milk in PBS at room temperature for 1 h, then was cut into individual lanes using scissors, and incubated with anti-SED mAb (negative control for Western blot) or anti-CD226 mAb FMU4 [18] at 4°C overnight. Then the membranes were washes six times with PBS-T and incubated with HRP labeled goat anti-mouse IgG secondary antibody (DAKO) at room temperature for 1 h. After four times washes with PBS-T, the individual membrane lanes were put together one by one and enhanced chemiluminescence (ECL) reagent (GE Healthcare) was applied to the membranes according to manufacturer's introductions, which were then exposed to an x-ray film (Kodak, Rochester, NY, USA).

### Statistical analysis

The Mann-Whitney *U *test or Kruskal-Wallis test was used to determine the significance of the differences of sCD226 and mCD226 expression between the groups. And the paired-samples T test was used to compare percentage of cytotoxicity between the groups. A *P *value < 0.05 was regarded as significant. Analysis was performed with SPSS (SPSS Inc., Chicago, IL, USA).

## Authors' contributions

ZWX, TZ, RZ, and YZ performed experiments and drafted the manuscript. WJ and CJS contributed materials to the study, helped to design the experiments and analyzed the data. KY and AGY participated in its design and helped to draft the manuscript. BQJ conceived of the study, and participated in its design and coordination and helped to draft the manuscript. All authors read and approved the final manuscript.
